# Supplementary material for: The EGFR-STYK1-FGF1 axis sustains functional drug tolerance to EGFR inhibitors in EGFR-mutant non-small cell lung cancer
Source: Cell Death Dis. 2022 Jul 15;13(7):611. doi: 10.1038/s41419-022-04994-4 (PMC9287553; doi:10.1038/s41419-022-04994-4)

Figure 2B - STYK1

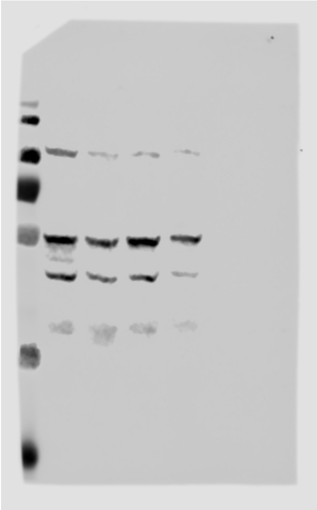

Figure 2B - ACTIN

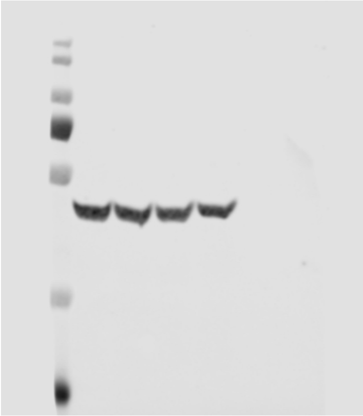

Figure 2E - STYK1

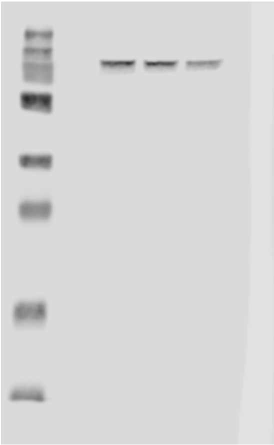

Figure 2E - ACTIN

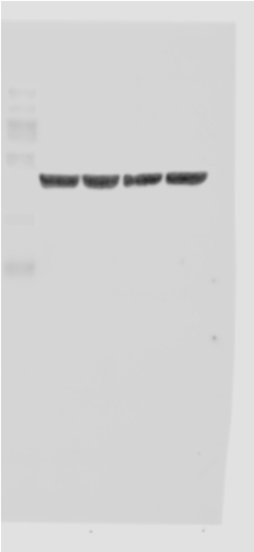

Figure 3C - STYK1

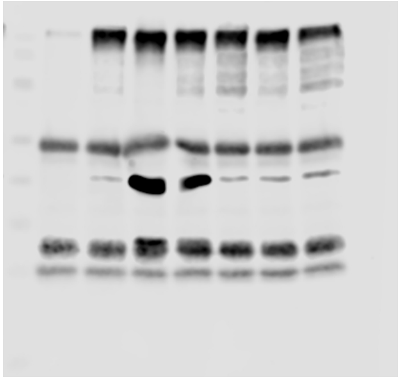

Figure 3C - EGFR

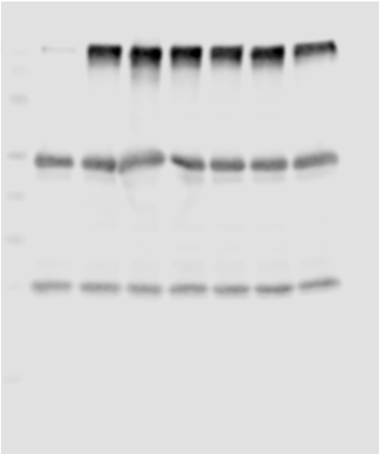

Figure 3C - STYK1 WCL

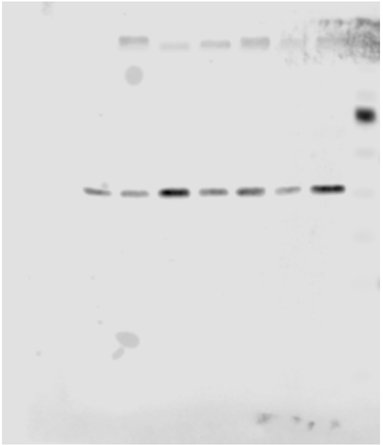

Figure 3C - EGFR WCL

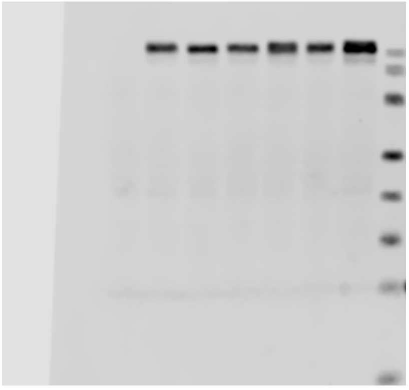

Figure 3C - ACTIN WCL

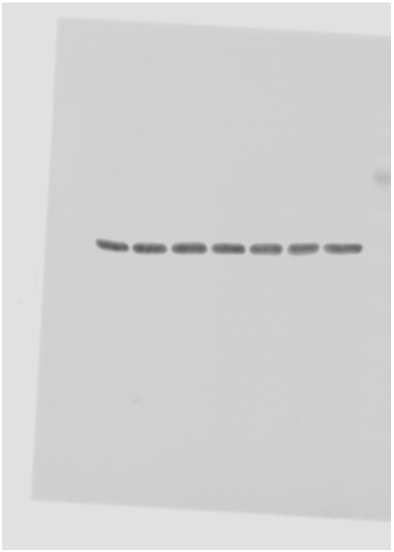

Figure 3D - STYK1

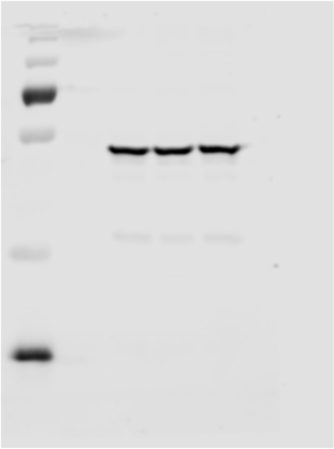

Figure 3D - EGFR

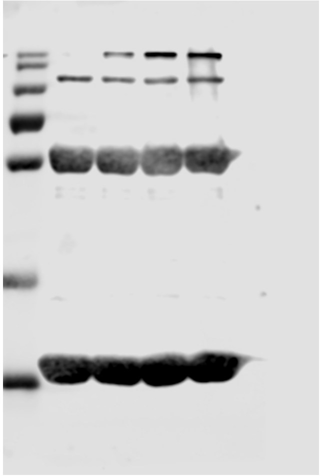

Figure 3D - STYK1 WCL

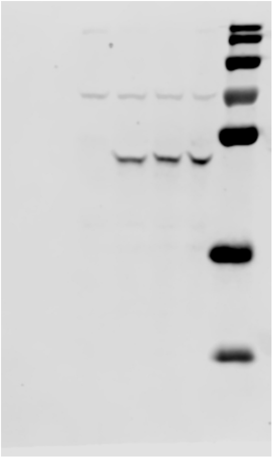

Figure 3D - EGFR WCL

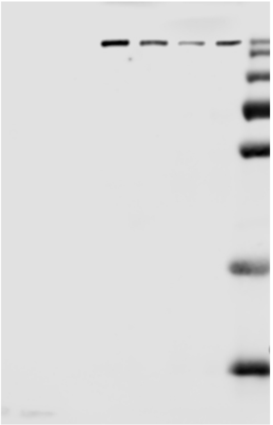

Figure 3D - ACTIN WCL

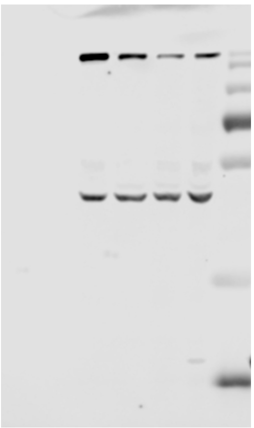

Figure 3E - STYK1

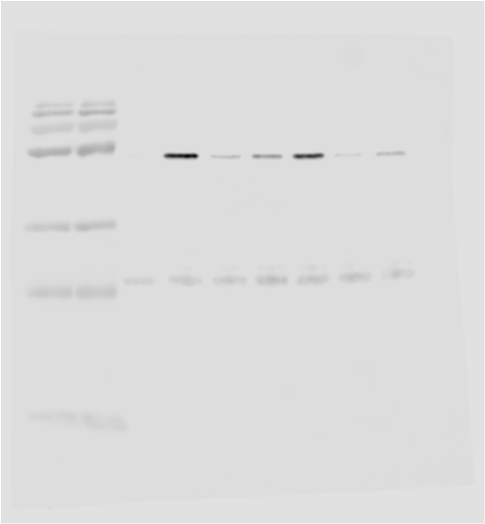

Figure 3E - EGFR

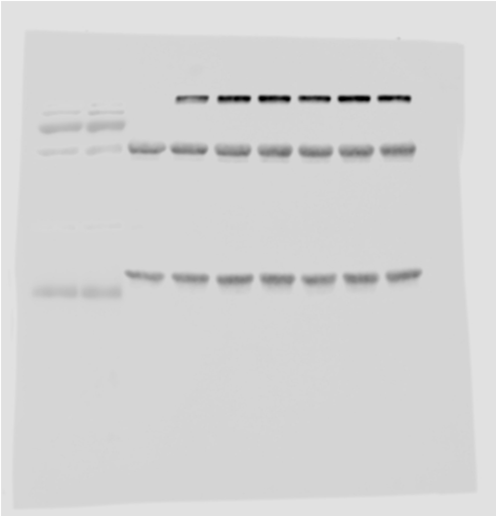

Figure 3E - STYK1 WCL

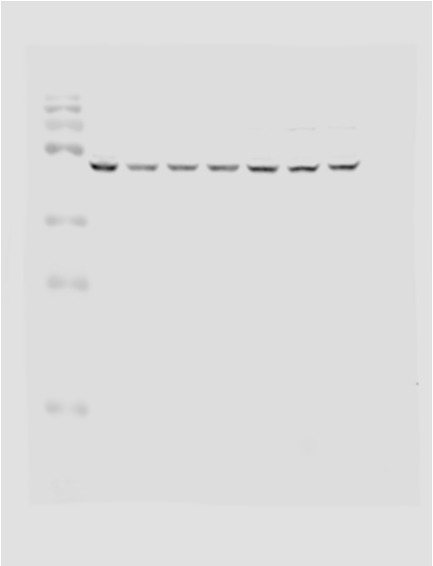

Figure 3E - EGFR WCL

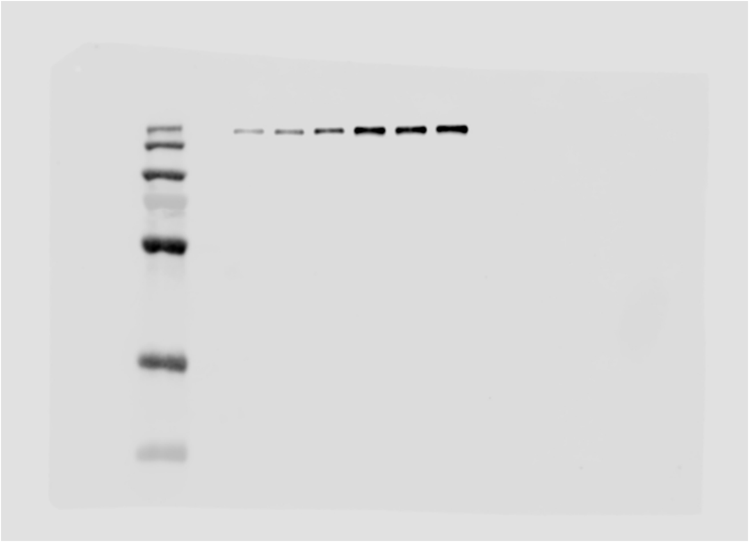

Figure 3E - pEGFR WCL

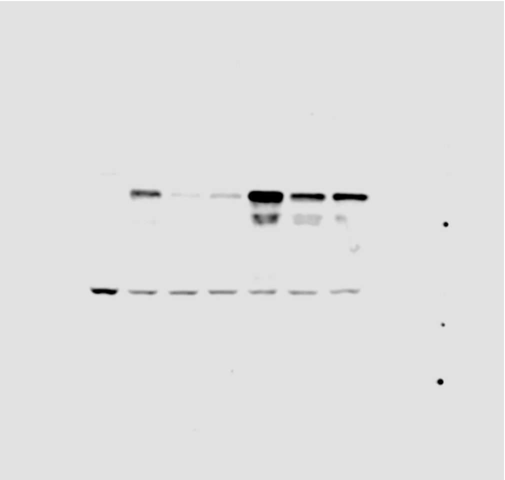

Figure 3E - Actin WCL

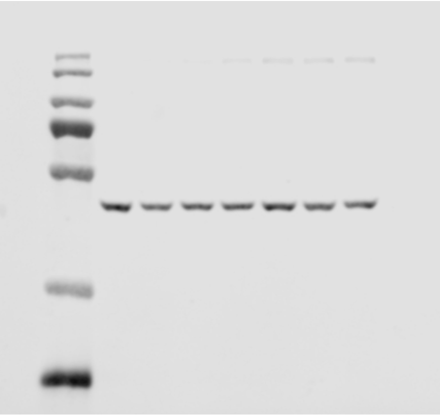

Figure 3F - STYK1

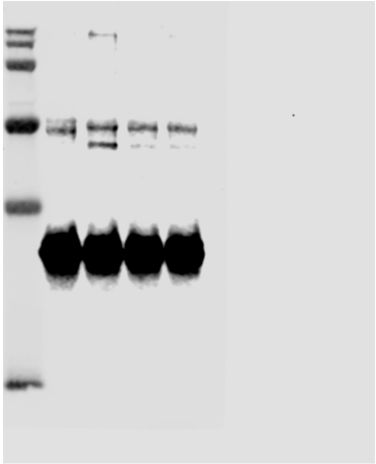

Figure 3F - EGFR

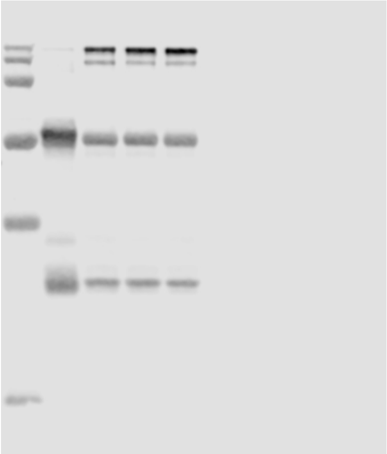

Figure 3F - pEGFR

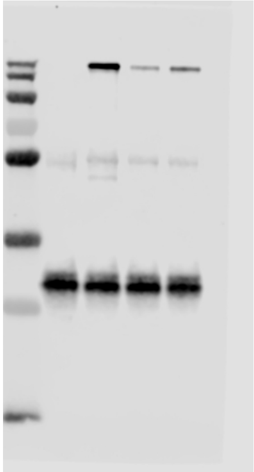

Figure 3F - STYK1 WCL

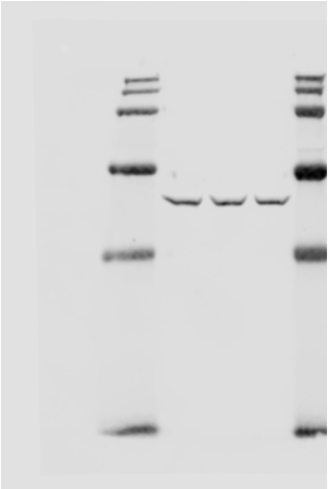

Figure 3F - EGFR WCL

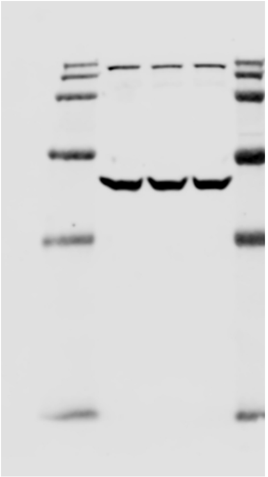

Figure 3F - pEGFR WCL

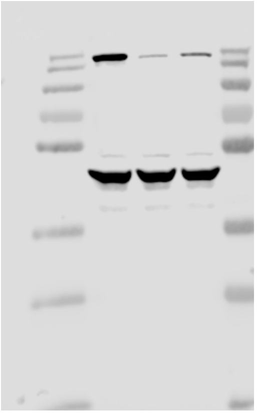

Figure 3F - Actin WCL

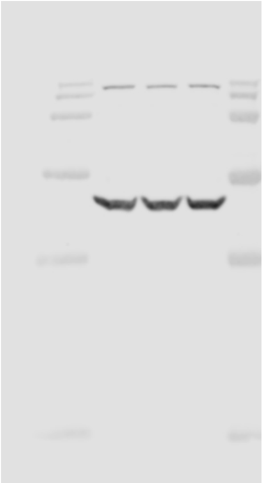

Figure 3G - STYK1

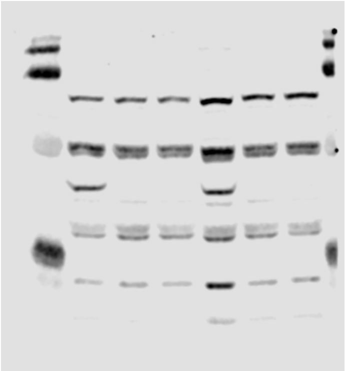

Figure 3G - EGFR

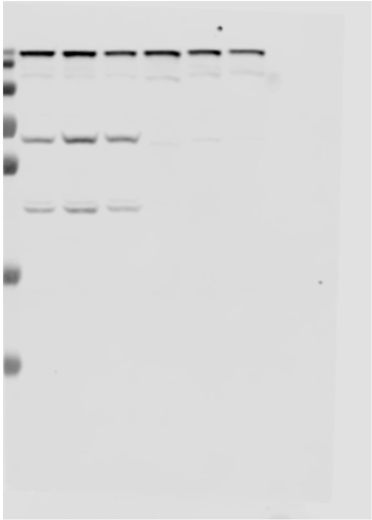

Figure 3G - pEGFR

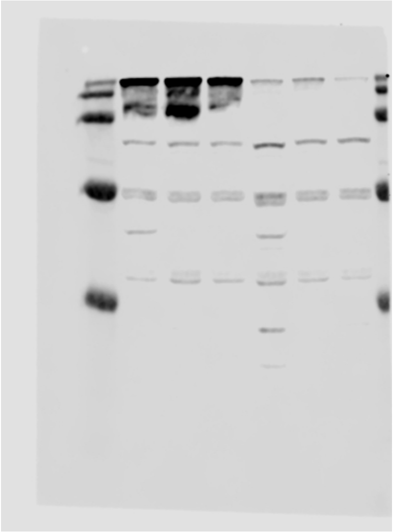

Figure 3G - pERK

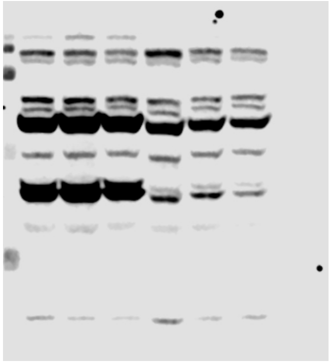

Figure 3G - ERK

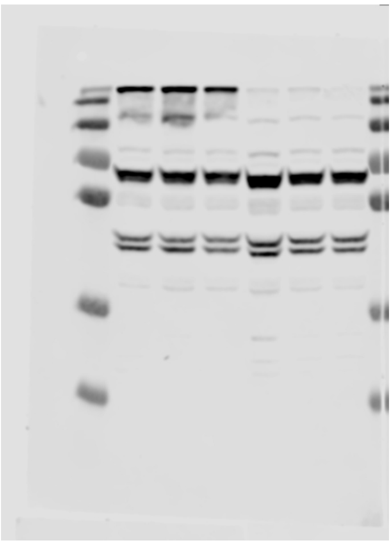

Figure 3G - pAkt

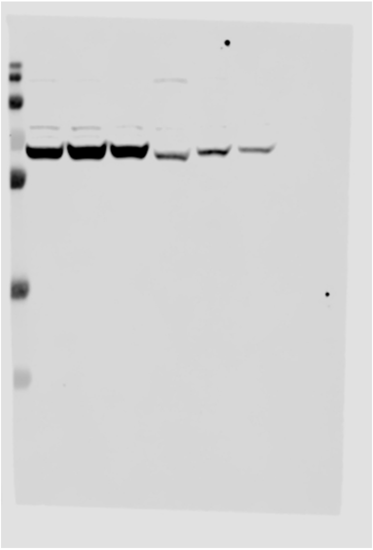

Figure 3G - Akt

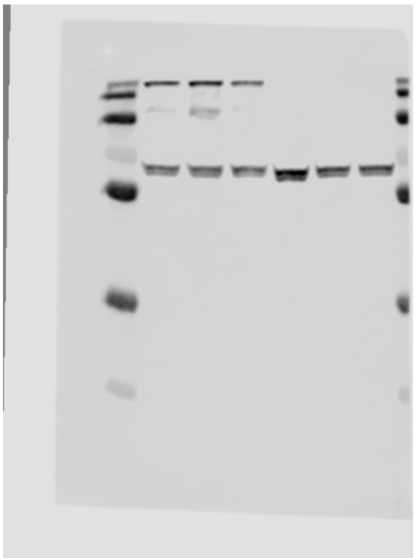

Figure 3G - Actin blot 1

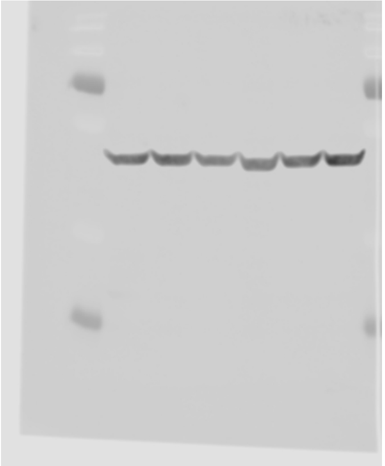

Figure 3G - Actin blot 2

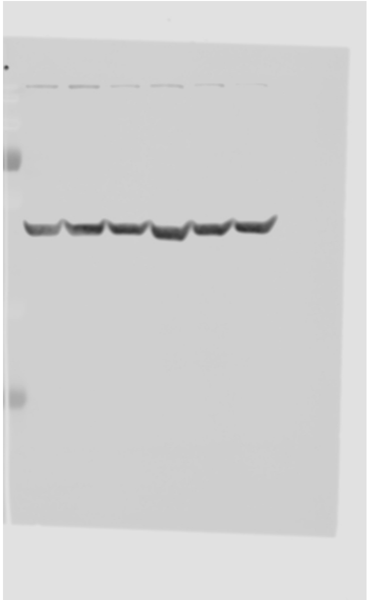

Figure S1B HCC827 - STYK1

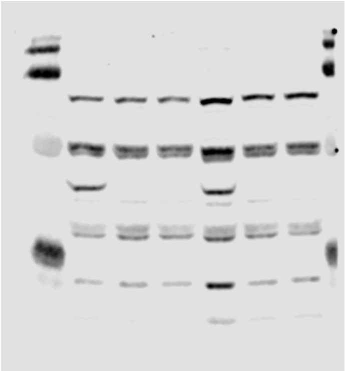

Figure S1B HCC827 - ACTIN

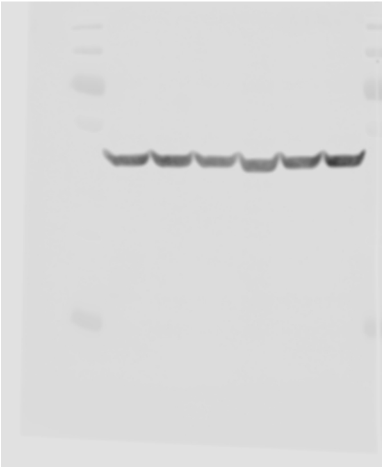

Figure S1B PC9 - STYK1

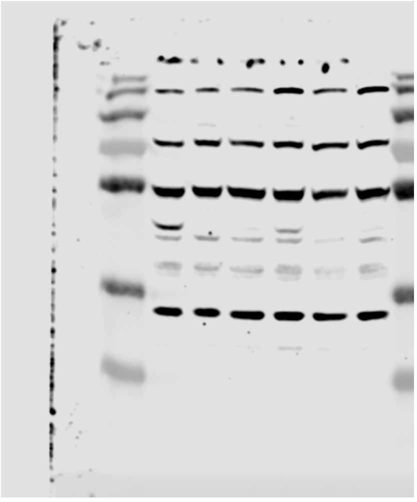

Figure S1B PC9 - ACTIN

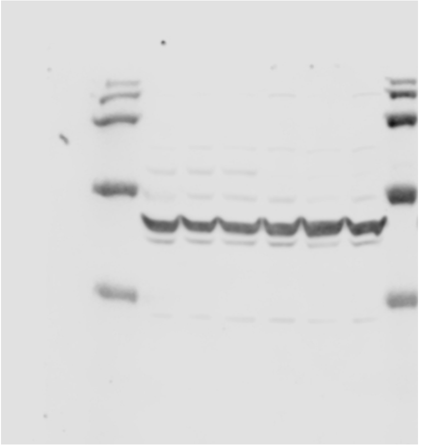

Figure S2A - STYK1

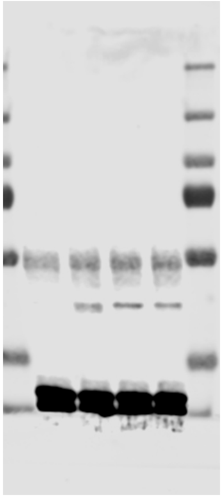

Figure S2A - EGFR

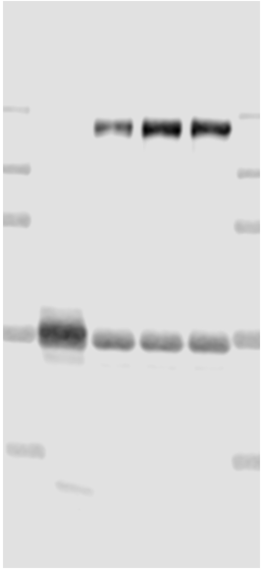

Figure S2A - pEGFR

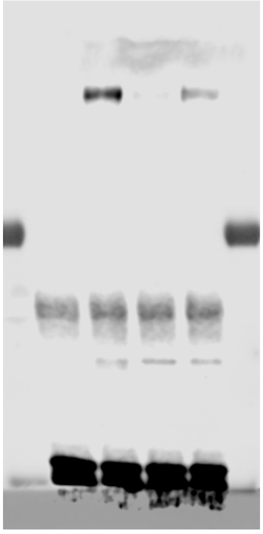

Figure S2A - STYK1 WCL

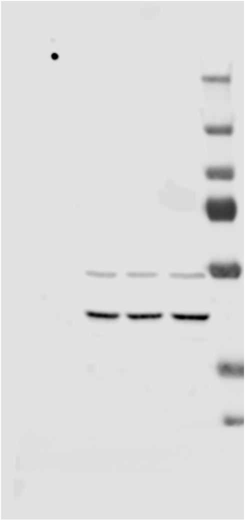

Figure S2A - EGFR WCL

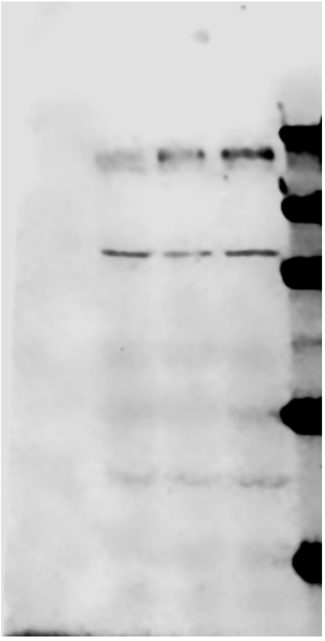

Figure S2A - pEGFR WCL

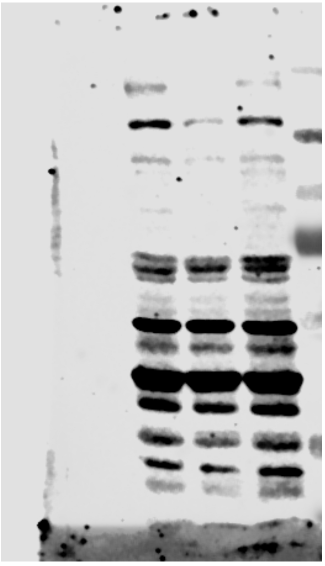

Figure S2A - ACTIN WCL

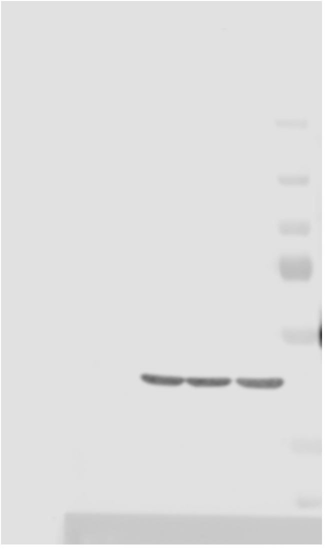

Figure S2B - STYK1

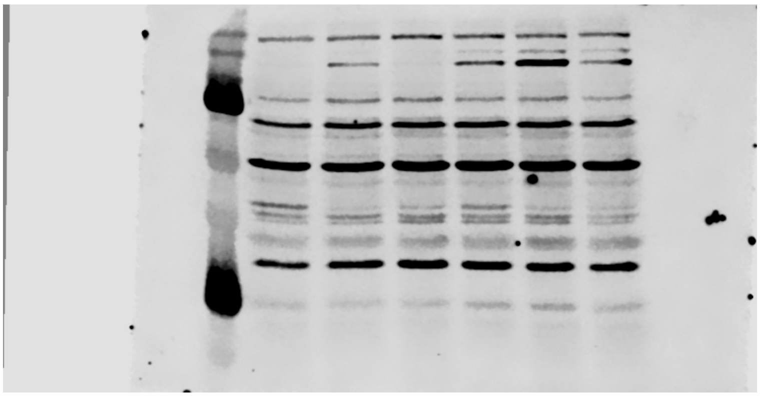

Figure S2B - EGFR

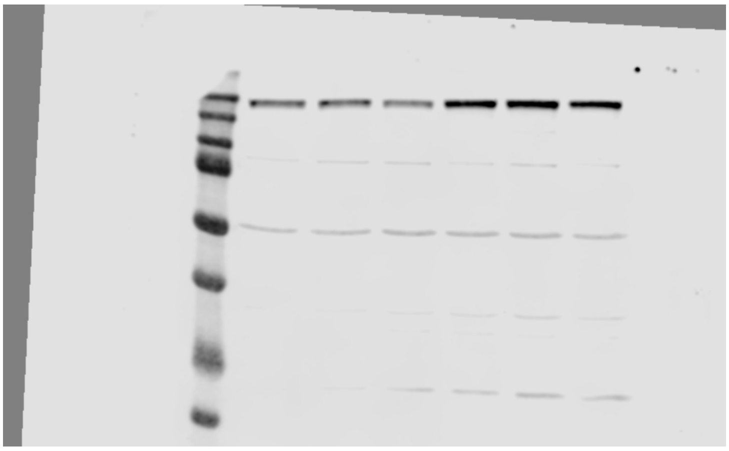

Figure S2B - pEGFR

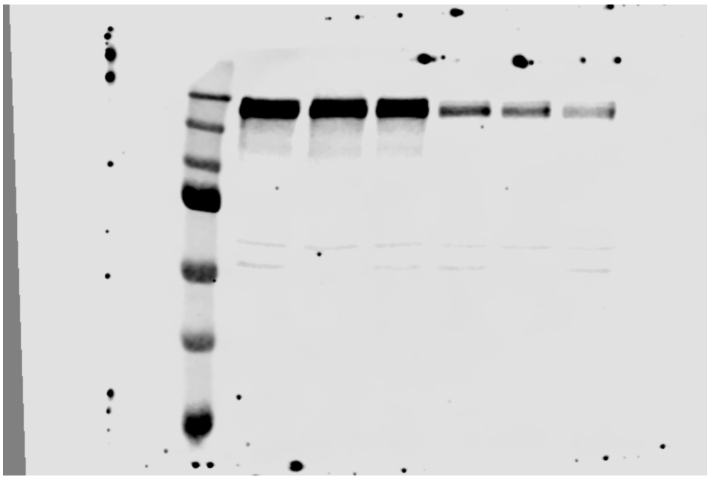

Figure S2B - pERK

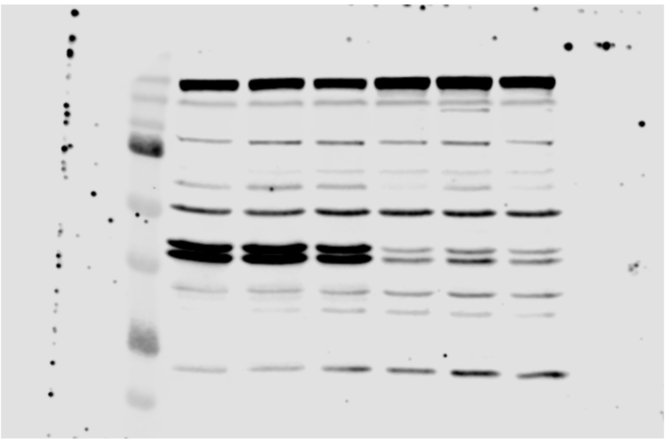

Figure S2B - ERK

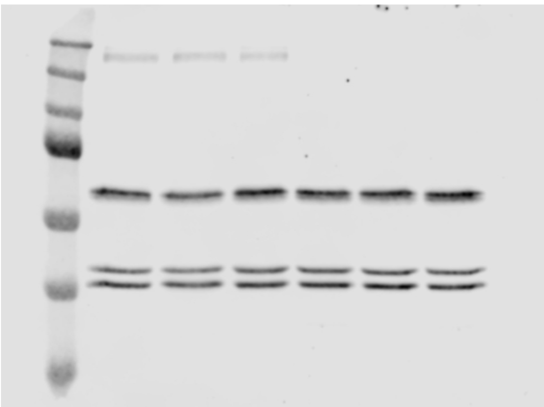

Figure S2B - pAKT

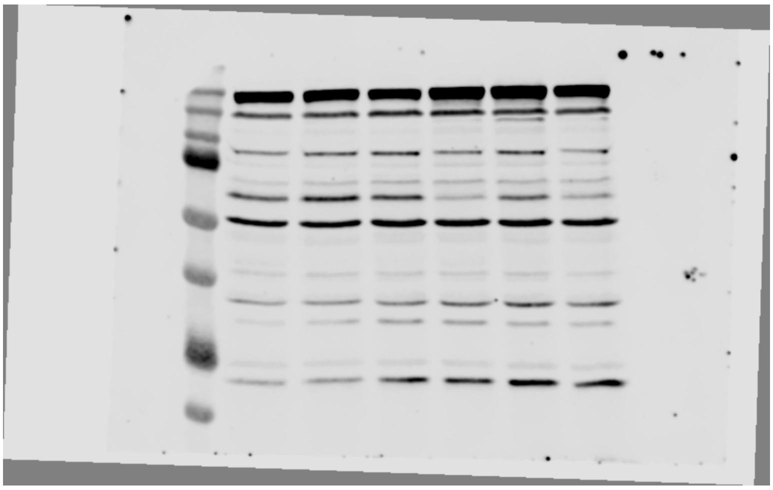

Figure S2B - Akt

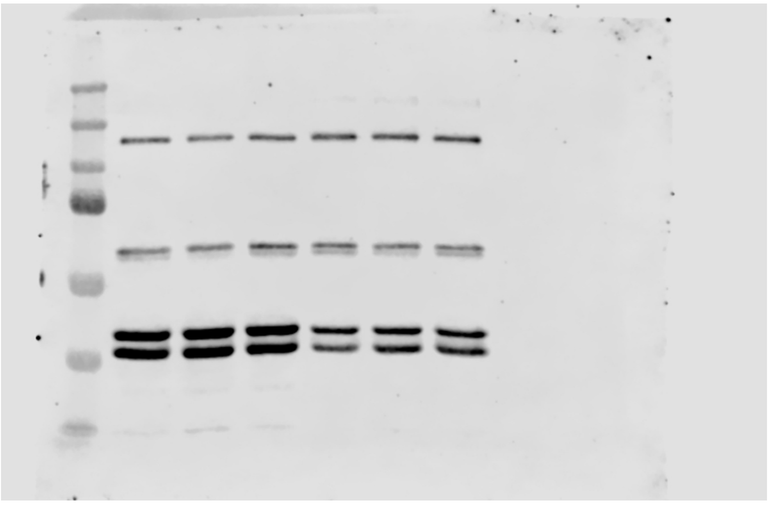

Figure S2B - Actin blot1

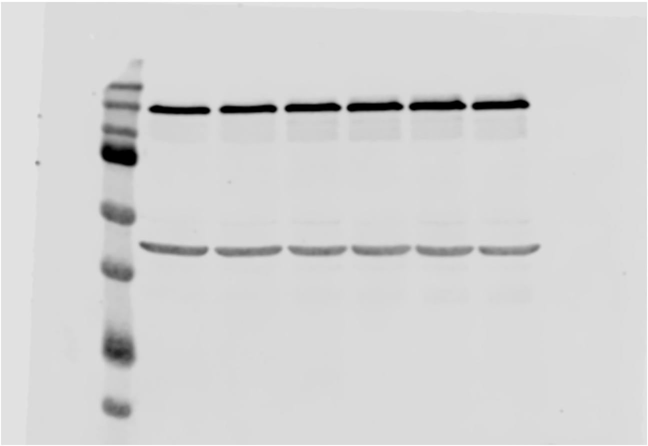

Figure S2B - Actin blot2

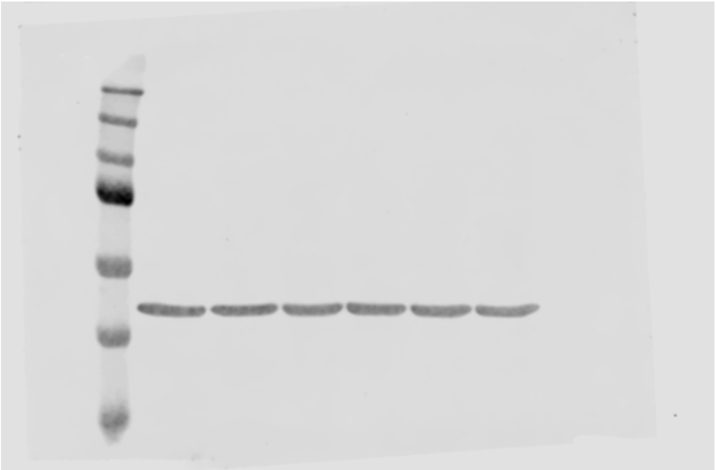

Supplement: Supplementary file 5 — Original western blots [file 41419_2022_4994_MOESM5_ESM.pdf]
